# Comparison of goat and cow milk-derived extracellular vesicle miRNomes

**DOI:** 10.1038/s41597-023-02347-0

**Published:** 2023-07-19

**Authors:** Zuzana Krupova, Christine Leroux, Christine Péchoux, Claudia Bevilacqua, Patrice Martin

**Affiliations:** 1 Université Paris-Saclay, INRAE, AgroParisTech, GABI, F-78350 Jouy-en-Josas, France; 2 Université Clermont Auvergne, INRAE, VetAgro Sup, UMR Herbivores, F-63122 Saint-Genès-Champanelle, France; 3 Department of Food Science and Technology, University of California Davis, Davis, CA USA

**Keywords:** miRNAs, RNA sequencing

## Abstract

miRNAs present in milk are mainly found in extracellular vesicles (EVs), which are nanosized membrane vesicles released by most of the cell types to ensure intercellular communication. The majority of the studies performed so far on these vesicles have been conducted on human and cow’s milk and focused on their miRNA content. The objectives of this study were to profile the miRNA content of purified EVs from five healthy goats and to compare their miRNome to those obtained from five healthy cows, at an early stage of lactation. EV populations were morphologically characterized using Transmission Electron Microscopy and Nanoparticle Tracking Analysis. The presence of EV protein markers checked by Western blotting and the absence of contamination of preparations by milk proteins. The size distribution and concentration of bovine and goat milk-derived EVs were similar. RNA-sequencing were performed, and all sequences were mapped to the cow genome identifying a total of 295 miRNAs. This study reports for the first-time a goat miRNome from milk EVs and its validation using cow miRNomes.

## Background & Summary

Milk is the main source of nutrients for newborn mammals, and is thought to play an important role in the development of the young immune system^1^. However, the mechanisms driving milk immune influence are still poorly understood and may be mediated by cells and bioactive molecules present in milk, including microRNAs (miRNAs). miRNAs are small non-coding RNAs (17–25 nucleotides long), highly conserved across species^2^, that post-transcriptionally regulate the expression of at least up to 60% of human genes, thus influencing many cellular processes, such as cell differentiation, proliferation, and cell death^3–5^. Milk miRNAs are, to some extent, packaged within extracellular vesicles (EVs) that are present in large amounts in milk^6,7^, and are, in such a way, protected from degradation in the gastrointestinal tract^6,8^. Therefore, they can survive digestion, be taken up by intestinal epithelial and immunologic cells in which they may be functionally active^9,10^. Thus, the survival of EV miRNAs and their influence on many biological processes make them crucial actors in the young and adult milk consumer health^8,10–12^. Whereas the repertories of miRNAs (miRNome) of human and cow’s milk EV were yet rather well described^13–16^, little is known in contrast on the miRNome of goat milk-derived EVs^17^. The objectives of this study were to determine the miRNome of milk-derived EVs from goat, and compare it with that of cow.

This study reports to our knowledge the first-time a goat miRNome from milk EVs and its validation using cow miRNomes. An average of 239 and 163 miRNAs were found with more than 1 Tag Per Million (TPM) in the cow and goat milk-derived EVs, respectively. The first 36 most abundant miRNAs present in goat milk-derived EVs accounted for 95% of TPM miRNAs. The miRNomes of the two ruminant species studied were very similar and among the TOP 10 most abundant miRNAs, 7, including the first 4 (*miR-148a*, *miR-21-5p*, *miR-26a* and *miR-30a-5p*) were common. These data were compared to published data on EVs from human and cow’s milk. Several abundant miRNAs were shared between species, such as *miR-148a*, *miR-21-5p*, *let-7a-5p*, *miR-200a-3p* and *miR-30d-5p*.

## Methods

### Materials

Since we did not perform any experiment on animal, no ethical approval was required for this study. Individual milk samples (100 mL each) were collected from five primiparous Alpine goats and five Holstein Friesian cows, at early lactation stages, 26 ± 9 days postpartum and 30 ± 7 days postpartum, respectively. Animals were reared in experimental farms of the Institut National de la Recherche pour l’Agriculture, l’Alimentation et l’Environnement (INRAE) at Domaine de Galle, 18520, Avord for goats, and at Domaine du Pin-au-Haras, 61310 Exmes, for cows. Preservatives [potassium dichromate: 0.05 g/L, 10 mM ε-amino caproic acid, and 10 μM phenylmethylsulfonyl fluoride (PMSF)] were added to milk immediately after milking to prevent proteolysis. Whole milk in 50 mL Falcon tubes were stored on ice during transport to the lab (1 hour). Depletion of somatic cells and milk fat was performed by centrifugation at 2,000 *g*, 15 min, 4 °C. Further, the second centrifugation of milk supernatant at 3,000 *g* for 30 min at 4 °C (Allegra X-15R, Beckman Coulter, France) was performed after transfer of liquid phase to a new tube with objective to eliminate the cell debris. The supernatant of skimmed and cell-free milk thus obtained was transferred to another 50 mL Falcon tubes and frozen at −80 °C before subsequent analysis.

### Isolation of bovine and goat milk-derived EVs using sucrose gradient ultracentrifugation

Isolation of milk-derived EVs as well as their validation and exo-RNA isolation were performed as follow. Skimmed milk samples stored previously at −80 °C were incubated in a water bath at 37 °C for 45 min. This step allows the reintegration of the free beta casein from the soluble phase into the casein micelles. The whey was obtained after acid precipitation by adding 10% (v/v) of 10% acetic acid, followed by incubation in a water bath at 37 °C for 10 min. Furthermore, an additional step specific for the treatment of goat milk characterized by a lower isoelectric point of caseins was performed by adding 1% (v/v) of 50% acetic acid to pre-treated goat milk samples. The mixture was incubated in water bath at 37 °C for 10 minutes. Coagulated milk samples were mixed shortly and 10% (v/v) of 1 M sodium acetate was added followed by incubation for 10 minutes at room temperature (RT). The major milk proteins (caseins) were pelleted by centrifugation at 1,500 *g*, 4 °C for 15 min. The whey fraction was filtered using vacuum-driven filtration system Steritop, 0.22 μm (Merck Millipore, France). Next, the cleared whey supernatants were concentrated using Amicon-15 100 kDa centrifugal filter units (Merck Millipore, France) at 4,000 *g* and 20 °C while obtaining the final volume of 6–8 mL. To pellet the EVs, the obtained concentrated retentate was ultra-centrifuged at 120,000 *g* for 1h10 at 4 °C using 50Ti rotor (Optima XPN-80, Beckman Coulter, France). The enriched EV’s pellets were resuspended in 500 μL of PBS 1x,then loaded at the top of 11 mL of pre-prepared sucrose gradient with decreasing sucrose concentration from 5% (on the top) to 45% (on the bottom) and ultra-centrifuged using SW41 rotor at 200,000 *g* for 18 h at 4 °C (Optima XPN-80, Beckman Coulter, France). Whole tube volume was fractionated into 1 mL fractions. The fractions 10–12 containing targeted EV populations were collected and diluted in 6 mL of PBS 1x,then centrifuged at 100,000 *g* using 50Ti rotor for 1h10 at 4 °C (Optima XPN-80, Beckman Coulter, France). The pellets were resuspended in 50 μL of PBS 1x. The resuspended pellets of fractions 10–12 were pooled and stored at −80 °C, until further analysis.

### Transmission electron microscopy (TEM) with uranyl acetate negative staining

The extracellular vesicles were analyzed after deposition of 4 µL of suspension on a 300-mesh copper grid covered with a carbon film membrane for 5 min, then stained with 1% uranyl acetate after absorption of the excess contrasting liquid and dried at room temperature. The grids were observed on a Hitachi HT7700 transmission electron microscope operated at 80 kVolts (Milexia). Images were acquired using a charge-coupled device camera (AMT).

### Nanoparticle Tracking Analysis (NTA) of milk-derived EVs by Nanosight NS300 (Malvern)

Particle concentration and size distribution were determined using a Nanosight NS300 instrument (Malvern, version NTA 3.2 Dev Build 3.2.16). The instrument was equipped with a Blue 405 nm laser, automatic syringe pump, sCMOS camera type and the NTA software v3.2. The capture settings were as follow: camera level of 12, Slider shutter of 1200, Slider gain of 146, and syringe pump speed of 50. Per sample, three videos of 90 seconds with a frame rate of 30 frames/sec were captured at constant acquisition temperature of 25 °C and subsequently analyzed with a threshold set up at 5. The NTA experiments were carried out with pre-diluted milk-derived EV samples in PBS 1x according to input sample concentrations, The obtained results were validated with at least 1,000 valid tracks for each triplicate.

### Bradford protein assay

One microliter of each EV sample was used to measure the protein concentration with a Bradford Protein Assay kit (Thermofisher). A standard linear curve was set up using BSA (Thermofisher). The protein quantification was achieved thanks to the reaction of proteins with a Coomassie Protein Assay reagent (Thermofisher). Absorbance measurement (OD) was performed in the visible spectrum at 595 nm on a spectrophotometer UVmini-1240 (Shimadzu).

### Western blot analysis

Western blotting (WB) was performed to validate the presence of known EV markers such as CD81 and hsp70 as well as to control the possible contamination by caseins. For this purpose, the presence of β-CN was also investigated. Twenty µg of EV proteins, corresponding approximately to a volume of 4-5 µL of bovine and goat EV samples were mixed with a denaturating solution of 4X Laemmli Buffer (Bio-Rad) and 10% β-mercaptoethanol prior to be heated for 10 min at 95 °C. Once cooled on ice, the protein samples were loaded onto a pre-cast 4–12% gradient polyacrylamide gel (Bio-Rad), and, after migration (15 mA/gel) in 1X TGS at 4 °C for 1 hour, subsequently transferred to a nitrocellulose membrane using Transblot Turbo system (Bio-Rad). The membrane was then blocked with 5% BSA + 0.05% Tween for 1 hour at room temperature. The membranes were thereafter exposed to anti-hsp70 and anti-CD81 rabbit primary antibodies (1:1000 in 5% BSA + 0.05% Tween, SBI) and to anti-β-casein rabbit polyclonal antibody (1:300 in 5% BSA + 0.05% Tween, Thermofisher) at 4 °C overnight. After incubation, several washing steps of the membranes with 0.05% Tween in TBS were applied. The membranes were subsequently incubated for one hour at room temperature with the goat anti-rabbit HRP secondary antibody (EXOAB-KIT, SBI) diluted 1:20000 in 5% BSA + 0.05% Tween TBS and washed. The chemiluminescence ECLPlus 2 kit (Pierce) was applied to the membranes 5 min prior imaging. Imaging was performed using ChemiDoc Touch Imaging System (Bio-Rad) equipped with CCD LAS1000 camera.

### RNA isolation

Total RNAs, including small RNAs were isolated from EV samples using an optimized mirVana Total *RNA Isolation* Kit (Life Technologies) with some modifications: firstly, Trizol LS Reagent (Ambion) was used for initial cell disruption instead of mirVana Phenol Lysis Reagent, followed by chloroform addition, ethanol precipitation (sample: 100% ethanol ratio of 1:1.25, v/v) and mirVana kit columns (Thermofisher) fractionation. Glycogen (1 µL per 800 µL of lysate) was added to improve RNA recovery. To obtain high quality RNA, a DNAse I treatment (Qiagen) was performed on columns for 15 min at RT according to manufacturer’s instructions. Finally, the RNAs were eluted in 50 µL of Elution buffer, then quantified by Bioanalyzer 2100 instrument (Agilent) using Pico chip and ND-1000 NanoDrop™ Spectrophotometer (Thermofisher).

### Small RNA library preparation and Next Generation RNA sequencing

MicroRNA NGS libraries were performed from 100 ng of total RNA using NEBNext library generation kit (New England Biolabs Inc.) according to the manufacturer’s instructions. Adaptors were ligated to the 3′ and 5′ of each RNA and converted into cDNA. A pre-amplification was carried out with specific primers for 15 cycles. After a purification using QiaQuick columns, the insert efficiency was evaluated on high sensitivity DNA chip using a Bioanalyzer 2100 instrument (Agilent). A size fractionation of obtained libraries was performed on a LabChip XT (Caliper Life Sciences) and bands corresponding to adaptors with 15–40 bp insert were excised using the manufacturer’s instructions. Sample quantifications were performed using qPCR. Based on quality and quantity of the inserts, the libraries were pooled in equimolar concentrations. An optimal concentration of the library pools determined by qPCR was used to generate the clusters on the flow cell surface before sequencing. Samples were sequenced on the Illumina NextSeq 500 system, according to the manufacturer instructions (Illumina). Sequencing data quality control (Q-score: QC), and adapter trimming were performed prior sequence mapping using miRbase 20. Because bovine is a phylogenetically related species of goat and has a higher number of miRNAs, the following results were based on the annotations obtained using *Bos taurus* as reference genome^18^.

Putative novel miRNAs were predicted from the sequences that did not map to any organism present in miRbase, or to other known RNA sequences. miRPara^19^ was used to analyze the potential folding of these sequences. These results were combined to identify 180 putative novel miRNAs (see raw data).

## Data Records

### Read data

The RNA-seq profiling was successfully completed for the 9 samples (one goat sample did not pass the RNA quality control). On average 11.6 million reads were obtained per sample with an average of 12.8 and 10.8 million of reads for *Capra hircus* and *Bos taurus* samples, respectively. The data were accessible with the GEO accession number: GSE227559^20^ and at Figshare number 21997106^21^.

### Identified microRNAs

**Fig. 1:**
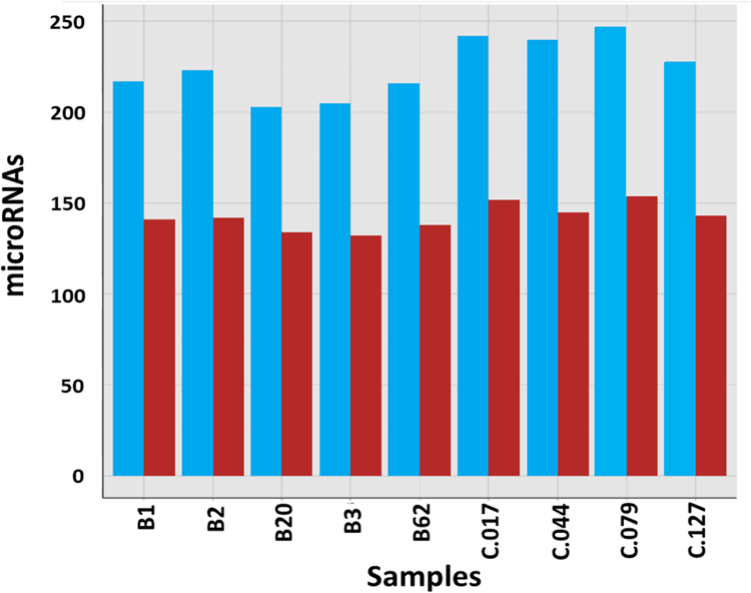
Number of identified known *bta*-miRNAs with number of counts (>1TPM per sample in blue bars and >10TPM per sample in red bars). Samples from bovine and caprine milk were identified as B and C, respectively.

After mapping the data on the bovine genome and counting to relevant entries in miRBase 20 the numbers of known microRNAs was determined. Taking into account all 9 samples (5 cows and 4 goats) of milk-derived EVs analyzed, a total of 295 miRNAs were identified. Goat samples displayed higher call-rates (number of identified known *bta*-miRNAs) than cow samples, ranging between 228 and 247, and 143 and 154 for number of counts > 1 TPM and > 10 TPM, respectively (Fig. 1).

### Most highly expressed miRNAs

In this study, the most widely represented miRNA (number one), with a mean TPM above 50,000 in both goats (88,925) and cows (123,755) is *miR-148a*. The four first miRNAs are the same in goat and cow, *miR-21-5p* at rank 2, positions 3 and 4 being reversed (*miR-26a* and *miR-30a-5p*). *miR-146b*, which ranks 5th in the goat species, is only 69th in the bovine species with a 150-fold lower level of expression (Table 1). The same is true for *miR-224* and *miR-378*, which rank 190 (*vs*. 36 in goats) and 53 (*vs*. 17 in goats) respectively, with expression levels 800 and 100 times lower in bovine than in caprine. The data are accessible with GEO accession number: GSE227559^20^ and on figshare^21^.

**Table 1:** Comparison between bovine and caprine of the first 36 most abundant goat milk-derived EV miRNAs (average TPM of the 4 individuals) that account for 95% of TPM miRNAs.

	Goat		miRNA	Cow	
	mean TPM	rank		rank	mean TPM
**>50,000**	**88,925**	**1**	**bta-miR-148a**	**1**	**123,755**
***>10,000***	***32,004***	***2***	***bta-miR-21-5p***	***2***	***29,604***
	***20,090***	***3***	***bta-miR-26a***	***4***	***18,977***
	***16,856***	***4***	***bta-miR-30a-5p***	***3***	***21,947***
	***14,286***	***5***	***bta-miR-146b***	69*	***101***
	***13,021***	***6***	***bta-let-7a-5p***	***5***	***17,518***
	**12*,535***	***7***	***bta-miR-30d***	***12***	***8,13*9**
	***11,977***	***8***	***bta-miR-200a***	***6***	**13*,66*8**
	***11,937***	***9***	***bta-miR-200b***	***10***	***9,353***
	***10,907***	***10***	***bta-let-7f***	***11***	***8,152***
	***10,108***	***11***	***bta-miR-200c***	***7***	***12,112***
*>1,000*	*6,061*	*12*	*bta-let-7b*	*9*	*9,915*
	*4,928*	*13*	*bta-miR-99a-5p*	*8*	*10,852*
	*4,807*	*14*	*bta-let-7g*	*13*	*3,860*
	*3,823*	*15*	*bta-let-7i*	*28*	*1,065*
	*3,372*	*16*	*bta-miR-22-3p*	*14*	*3,689*
	*3,318*	*17*	*bta-miR-378*	53*	318
	*2,741*	*18*	*bta-miR-*19*1*	*19*	*1,873*
	*2,735*	*19*	*bta-miR-375*	*20*	*1,815*
	*2,611*	*20*	*bta-miR-92a*	*16*	*2,581*
	*2,179*	*21*	*bta-miR-151-3p*	*22*	*1,591*
	*2,143*	*22*	*bta-miR-423-3p*	*27*	*1,205*
	*1,845*	*23*	*bta-miR-423-5p*	*26*	*1,290*
	*1,834*	*24*	*bta-miR-27b*	*15*	*3*,25*0*
	*1,812*	*25*	*bta-miR-103*	*31*	*966*
	*1,575*	*26*	*bta-miR-181a*	*36*	*706*
	*1,543*	*27*	*bta-miR-186*	*21*	*1,717*
	*1,526*	*28*	*bta-miR-101*	*23*	*1,567*
	*1,435*	*29*	*bta-miR-320a*	*17*	*2,055*
	*1,415*	*30*	*bta-miR-26b*	*18*	*1,961*
	*1,059*	*31*	*bta-miR-*30*e-5p*	*30*	*1,038*
	*1,035*	*32*	*bta-miR-30c*	46*	*406*
	*1,031*	*33*	*bta-let-7c*	*24*	*1,542*
<1,000	934	34	bta-miR-27a-3p	33	791
	827	35	bta-miR-532	*25*	1,315
	815	36	bta-miR-224	190*	1

*Highlight values corresponding to higher ranks than 36 TPM in cow samples. The bold, bold italics, italics and standard depict the miRNAs detected with more than 50,000, between 50,000 and 10,000, between 1,000 and 10,000, and less than 1,000 TPM in goat, respectively.

## Technical Validation

### Morphological characterization of EVs populations

#### Transmission electron microscopy (TEM)

**Fig. 2:**
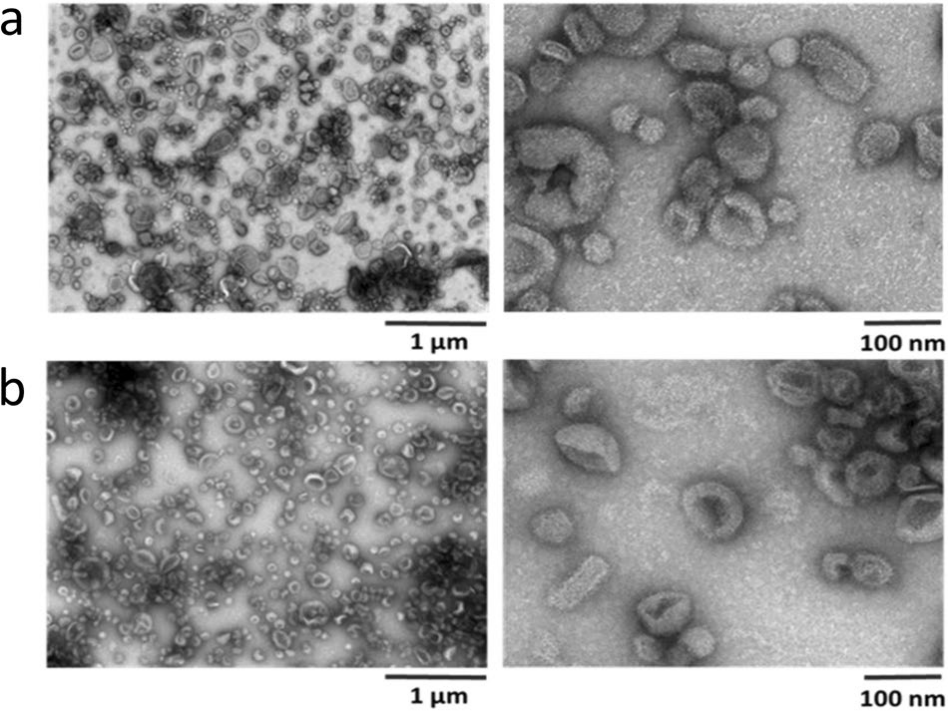
Transmission Electron Microscopy (TEM) with negative staining using uranyl acetate of bovine (**a**) and goat (**b**) milk-derived extracellular vesicles.

As shown in Fig. 2, milk-derived EVs appeared rather homogeneous in shape, in cow milk (A) as well as in goat milk (B), ranging in size between 30 and 150 nm. They were sometimes clustered in aggregates. Even though samples must be dehydrated before analysis no cell debris and minimal background were observed, revealing the efficiency of the purification procedure.

#### Nanoparticle tracking analysis (NTA) of EVs

**Fig. 3:**
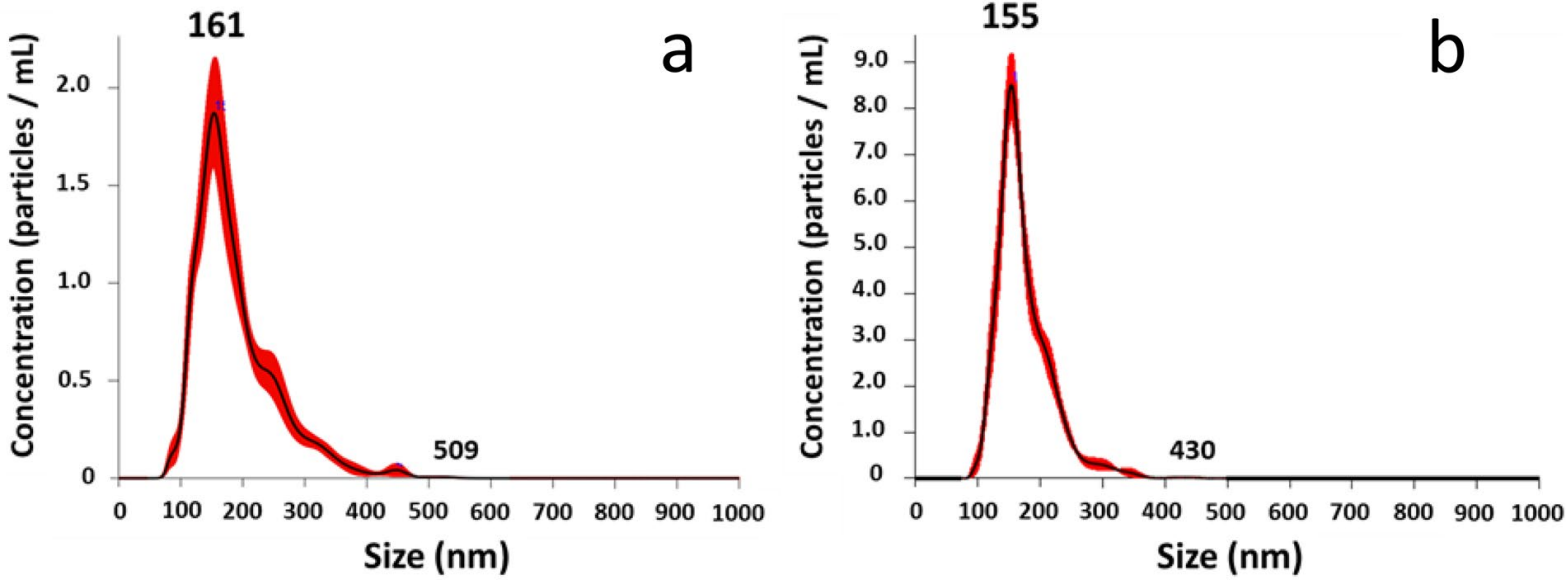
Typical profiles of size distribution evaluated by Nanosight S300 NTA (Malvern) of bovine (**a**) and goat (**b**) milk-derived extracellular vesicles. These profiles correspond to triplicate analyses of individual samples.

Based on the NTA data obtained using Nanosight NS300 (Malvern), the average diameter of milk-derived EV (±standard error) was 165.00 ± 3.44 nm for cows, and 169.66 ± 3.65 nm for goats (Table 2). The distribution of mEV populations was “gaussian-like” for both species with a quite unique peak and a narrow range (Fig. 3), revealing a homogeneous population of particles both within and between species.

**Table 2:** Average size distribution and concentration of bovine and goat milk-derived extracellular vesicles determined from Nanosight S300 (Malvern) NTA data.

	Bovine milk-derived EVs		Goat milk-derived EVs	
	average	sd	average	sd
**Size distribution**	165.00	3.44	169.66	3.65
**Measured particle concentration of isolated EV population (nb of particles/mL)**	3.73E + 12	1.29E + 12	1.76E + 12	6.17E + 11
**Calculated particle concentration relative to 1 mL of milk (nb of particles/mL)**	8.28E + 09	2.87E + 09	3.92E + 09	1.37E + 09
**Protein concentration (µg/µL)**	6.63	0.39	4.10	0.61

The average protein concentration was estimated using Bradford Protein Assay.

### Western blot analysis of proteins from EVs preparations

**Fig. 4:**
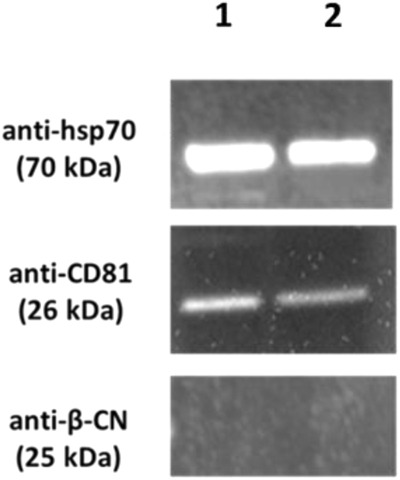
Western blot analysis of EV-associated markers and casein contaminant.

The presence of EV-associated markers (hsp70 and CD81) and the absence of milk protein contaminant were clearly demonstrated by Western Blotting (Fig. 4) both for EVs isolated from cow’s milk (1) and from goat’s milk (2).

### Quality control of RNA samples

**Fig. 5:**
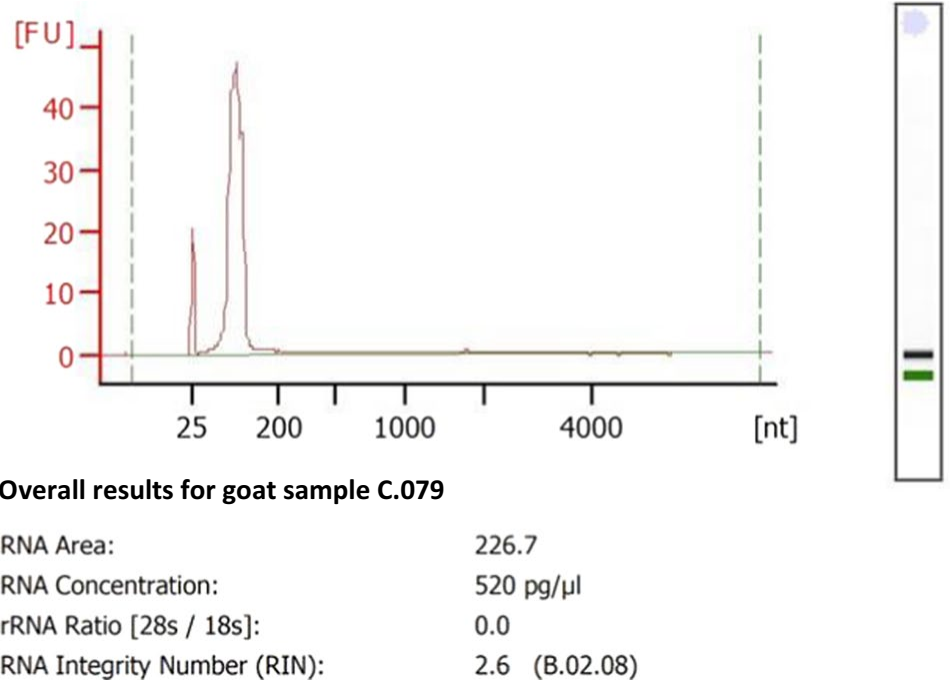
Gel-like image and electropherograms obtained with 1/10^th^ diluted RNA sample of goat milk-derived EVs.

The Bioanalyzer RNA assay providing an objective measurement of RNA preparations quality and a sensitive analysis to resolve nucleic acid samples in the size range of 10 to 4000 nt was performed. However, it must be kept in mind that the RNA Integrity Number (RIN) is meaningless as long as the RNA preparation does not contain ribosomal RNA, which is an additional proof of the quality of the miRNA preparations. A typical result of quality control from goat milk-derived EV is given in Fig. 5.

### Validation of miRNA sequencing

#### Principal component analysis plot

**Fig. 6:**
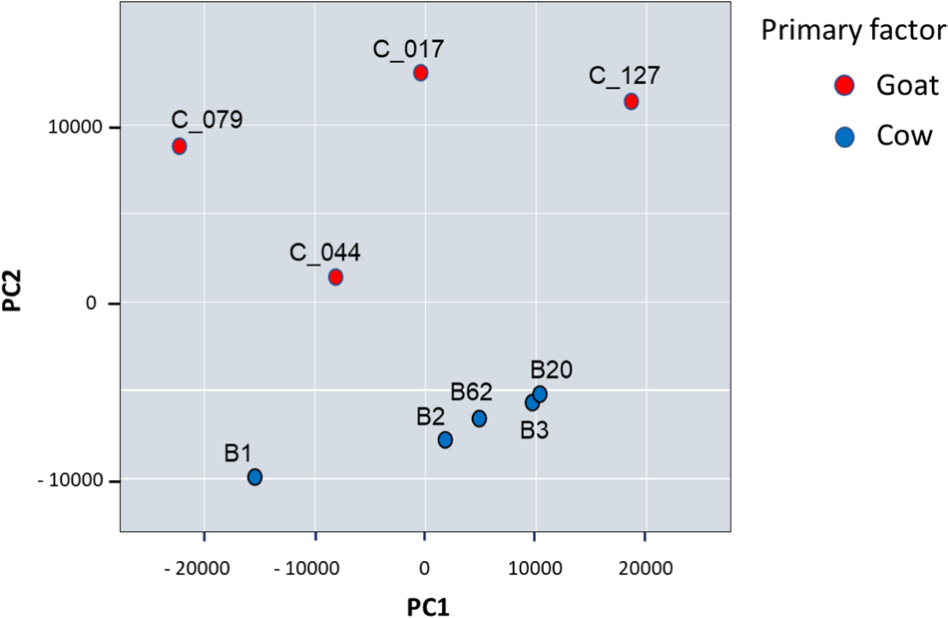
Principal component analysis plot. The PCA was performed on all samples passing QC (4 goat and 5 cow samples) using the TOP 50 miRNAs with highest CV (based on TPM normalized reads).

Principal Component Analysis (PCA) was used to explore the naturally arising sample classes based on the expression profile. Here, by including the TOP 50 miRNAs that had the largest variation across all samples, an overview of how the samples cluster based on this variance was obtained. The data was normalized with tag per million (TPM) method and converted to a log2 scale. Then all features were filtered on “expressed in all samples” criteria and the 50 features with the highest coefficient of variation (% CV) selected for the analysis. The largest component in the variation is plotted along the X-axis and the second largest is plotted on the Y-axis. As shown in Fig. 6, the samples tend to cluster based on the organism (mainly along the second axis, *i.e*. second component in the variation), the first factor of variation being the individual intra-species variability (X-axis). Thus, PCA separated the samples according to their origin.

#### Heat map and unsupervised clustering

**Fig. 7:**
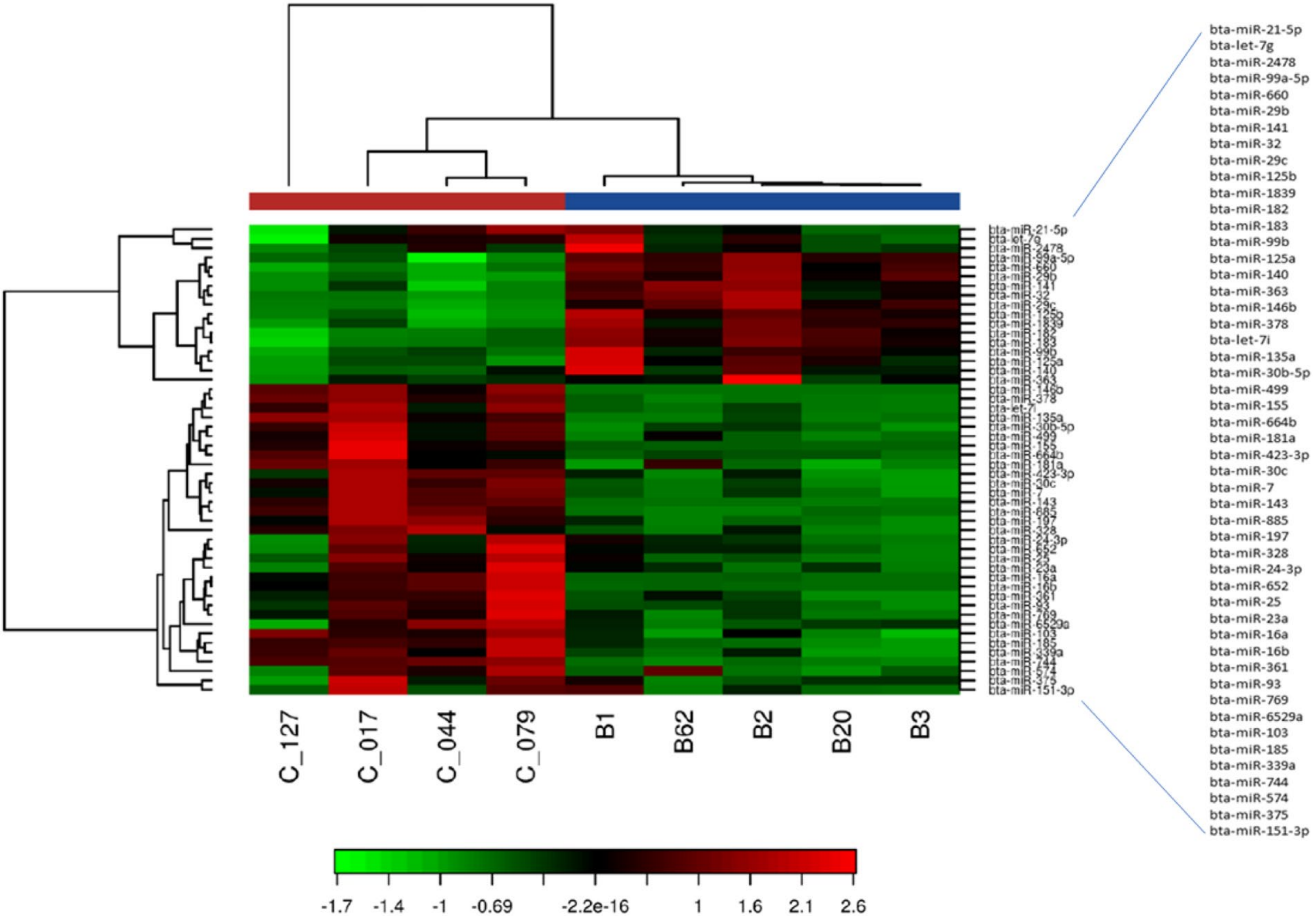
Heat Map and unsupervised hierarchical clustering using each 4 goat and 5 cow samples and the TOP 50 microRNAs with highest CV based on TPM normalized counts. Red and green color represent the expression level above and below the mean, respectively.

The heat map diagram shows the result of the two-way hierarchical clustering of miRNAs and samples (Fig. 7). The data is normalized with TPM method and converted to a log2 scale. Then all features are filtered on “expressed in all samples” criteria and the 50 miRNAs with the highest coefficient of variation (%CV) selected for the analysis. Each row represents one miRNA, and each column represents one sample. The color of each point represents the relative expression level of a miRNA across all samples: This analysis led to classifying the samples according to their organism of origin.

For goats, the 10 most abundant miRNAs account for nearly 75% (ranging between 72,7 and 73,8%) of the 295 miRNAs mapped, at least in one sample, referring to the bovine genome. For cows, the 10 most abundant miRNAs account for nearly 80% (ranging between 78,8 and 80,0%) of the miRNAs mapped. The most abundant miRNAs in both species is *miR-148a*, which occupies almost systematically the pole position in milk-derived miRNAs profiling studies, whatever the mammalian species considered, including human^22,23^ and pig^24^. Our results are validated by the comparison with data previously reported in cow and human, showing a close pattern of abondance (Table 3). The miRNAs, which were not in the TOP 10 miRNAs of the present study, were detected with a lower rank but were present.

**Table 3:** The TOP 10 miRNAs in goat, cow milk-derived extracellular vesicles obtained in the present study (a) compared with cow (b)^25^ and human (c^22^; d^23^) data.

rank	Goat^a^	Cow^a^	Cow^b^	Human^c^	Human^d^
1	***miR-148a***	***miR-148a***	***miR-148a-3p***	***miR-148a-3p***	***miR-30d-5p***
2	***miR-21-5p***	***miR-21-5p***	***let-7a***	miR-22-3p	***miR-148a-3p***
3	*miR-26a*	*miR-30a-5p*	let-7b	***miR-30d-5p***	***miR-200a-3p***
4	***miR-30a-5p***	*miR-26a*	***miR-21-5p***	let-7b-5p	miR-200c-3p
5	miR-146b	***let-7a-5p***	miR-99a-5p	***miR-200a-3p***	***let-7a-5p***
6	***let-7a-5p***	***miR-200a***	***let-7f-5p***	***let-7a-5p***	***miR-200b-3p***
7	***miR-30d***	miR-200c	let-7c	***let-7f-5p***	***miR-21-5p***
8	***miR-200a***	miR-99a-5p	miR-200c	miR-146b-5p	let-7b-5p
9	***miR-200b***	let-7b	*miR-26a-5p*	miR-24-3p	*let-7f-5p*
10	***let-7f***	***miR-200b***	***miR-30d***	***miR-21-5p***	***miR-30a-5p***

The bold italics depict the miRNAs present at least once in the TOP10 of each species, underlined italics those present in ruminant species.

## Data Availability

No custom code was written.
